# Time to eligibility for antiretroviral therapy in adults with CD4 cell count > 500 cells/μL in rural KwaZulu-Natal, South Africa

**DOI:** 10.1111/hiv.12255

**Published:** 2015-05-11

**Authors:** N McGrath, RJ Lessells, ML Newell

**Affiliations:** 1 Academic Unit of Primary Care and Population Sciences, University of SouthamptonSouthampton, UK; 2 Department of Social Statistics and Demography, University of SouthamptonSouthampton, UK; 3 Africa Centre for Health and Population Studies, University of KwaZulu-NatalMtubatuba, South Africa; 4 Department of Clinical Research, London School of Hygiene and Tropical MedicineLondon, UK; 5 Academic Unit of Human Development and Health, University of SouthamptonSouthampton, UK

**Keywords:** antiretroviral therapy, HIV, sub-Saharan Africa

## Abstract

**Objectives:**

Understanding of progression to antiretroviral therapy (ART) eligibility and associated factors remains limited. The objectives of this analysis were to determine the time to ART eligibility and to explore factors associated with disease progression in adults with early HIV infection.

**Methods:**

HIV-infected adults (≥ 18 years old) with CD4 cell count > 500 cells/μl were enrolled in the study at three primary health care clinics, and a sociodemographic, behavioural and partnership-level questionnaire was administered. Participants were followed 6-monthly and ART eligibility was determined using a CD4 cell count threshold of 350 cells/μl. Kaplan − Meier and Cox proportional hazard regression modelling were used in the analysis.

**Results:**

A total of 206 adults contributed 381 years of follow-up; 79 (38%) reached the ART eligibility threshold. Median time to ART eligibility was shorter for male patients (12.0 months) than for female patients (33.9 months). Male sex [adjusted hazard ratio (aHR) 3.13; 95% confidence interval (CI) 1.82–5.39], residing in a household with food shortage in the previous year (aHR 1.58; 95% CI 0.99–2.54), and taking nutritional supplements in the first 6 months after enrolment (aHR 2.06; 95% CI 1.11–3.83) were associated with shorter time to ART eligibility. Compared with reference CD4 cell count ≤  559 cells/μl, higher CD4 cell count was associated with longer time to ART eligibility [aHR 0.46 (95% CI 0.25–0.83) for CD4 cell count 560–632 cells/μl; aHR 0.30 (95% CI 0.16–0.57) for CD4 cell count 633–768 cells/μl; and aHR 0.17 (95% CI 0.08–0.38) for CD4 cell count > 768 cells/μl].

**Conclusions:**

Over one in three adults with CD4 cell count > 500 cells/μl became eligible for ART at a CD4 cell count threshold of 350 cells/μl over a median of 2 years. The shorter time to ART eligibility in male patients suggests a possible need for sex-specific pre-ART care and monitoring strategies.

## Introduction

By the end of 2012, in sub-Saharan Africa, an estimated seven million people of 25 million living with HIV received antiretroviral therapy (ART) [1], but most HIV-infected people have either not yet accessed or are not yet eligible for ART. With increasing numbers of people diagnosed early in the course of infection, there is a need for evidence-based care strategies for this group [1].

Studies in different settings have estimated the time from HIV-1 seroconversion to reaching a CD4 cell count threshold of 350 cells/μl for treatment eligibility to be 4–5 years [2],[3]. Virological and immunological factors associated with disease progression have been well characterized [4], but the impact of sociodemographic and psycho-behavioural factors on disease progression and time to ART eligibility has been studied less extensively, particularly in sub-Saharan Africa [5],[6]. Understanding the influence of these factors on HIV disease progression is important to inform pre-ART care and monitoring strategies, and for policy decisions concerning the timing of ART initiation and the use of ART for prevention. There are few data on time to ART eligibility in those who present in early HIV infection, although increasing numbers of people are diagnosed and enter care at higher CD4 cell counts [7]. We used data from a longitudinal study investigating the impact of ART on the partnerships and sexual behaviour of HIV-infected individuals to explore factors associated with disease progression in adults in the early stages of HIV infection [8].

## Methods

People can access HIV counselling and testing at any primary health care (PHC) clinic in the Hlabisa sub-district [9]. Blood is taken for CD4 cell count measurement immediately after a positive HIV diagnosis. The South African guidelines for ART eligibility have changed over the course of this study: CD4 cell count < 200 cells/μl or World Health Organization (WHO) stage 4 until March 2010; CD4 cell count cut-off < 350 cells/μl for pregnant women and individuals with active tuberculosis (TB) disease from April 2010 to August 2011; < 350 cells/μl for all from August 2011.

Adult (≥ 18 years old) HIV-infected men and nonpregnant women accessing care at three of the PHC clinics and resident within the Africa Centre Demographic Surveillance Area were potentially eligible for inclusion in the parent study and were approached for recruitment if their CD4 cell count was < 200 cells/μl or > 500 cells/μl [8]. Individuals were asked to consent separately to linkage of their study data with routine clinical data collected in the HIV programme, specifically clinic attendance history and laboratory test results, and with the Africa Centre Demographic Information System (ACDIS) database for their residential history. Sociodemographic, behavioural and partnership-level data were collected at enrolment [10]. Study participants were interviewed 6-monthly during routine clinic visits, or where necessary during home visits or by phone.

The study was approved by the ethics committees of the University of KwaZulu-Natal (BF083/08), the London School of Hygiene and Tropical Medicine (5413), and the KwaZulu-Natal Department of Health.

Individuals with CD4 cell count > 500 cells/μl at enrolment were advised to return 6-monthly for repeat clinical assessment and CD4 cell count measurement; those with at least one post-enrolment CD4 cell count result contributed to the analyses. Characteristics at enrolment for individuals included in the analyses were compared with those for individuals who were not using χ^2^ tests for categorical variables and Wilcoxon rank sum tests for continuous variables.

Study recruitment started in January 2009, with follow-up for 36 months maximum. While many individuals continued to access care from the local HIV programme, with additional post-study CD4 cell count results, only results from tests conducted during study follow-up were included in this analysis.

Individuals who initiated ART for clinical reasons were also considered ART-eligible; their ART initiation date was their eligibility date. ART initiation date was documented during follow-up or by linkage to the HIV programme database. Follow-up time for individuals not meeting ART eligibility criteria was censored at the last CD4 cell count test date.

Time from study enrolment to ART eligibility, defined as CD4 cell count < 350 cells/μl, was estimated by Kaplan − Meier survival analysis. Associations between the time to ART eligibility and sociodemographic and behavioural factors at enrolment, including ART knowledge, social capital, social support, relationship characteristics, and internalized HIV stigma (composite score from adapted scales), were initially explored using univariable Cox models [8,10]. We considered all variables that were significant at univariable level (p value < 0.05) for the multivariable model. Given previously reported faster disease progression in men, we explored an interaction term between CD4 cell count and sex in the final multivariable model. CD4 cell count was included in the model as a set of binary indicators representing quartiles of the baseline distribution: ≤ 559, 560–632, 633–768 and > 768 cells/μl. The interaction between CD4 cell count and sex was tested using seven binary indicators to represent the eight possible categories. A likelihood ratio test of this interaction was performed and the results compared to a χ^2^ distribution with three degrees of freedom. The final model was tested to check that the proportional hazards assumption held.

## Results

Overall, 247 adults had been enrolled by March 2011; 41 (17%) had no post-enrolment CD4 cell count result and did not contribute to the analyses. These 41 did not differ from the 206 included by age (*P* = 0.46), sex (*P* = 0.56) and enrolment CD4 cell count (*P* = 0.69). Thus, 206 adults (14% male; median age 34 years) contributed 381 years of follow-up [median 24 months; interquartile range (IQR) 15–30 months] (Table 1), with a median number of CD4 cell count measurements of 3 (IQR 2–4). CD4 cell count measurement number was not significantly associated with any factor included in the final model (data not shown). Overall, 79 (38%) participants became eligible for ART: 75 by CD4 cell count criteria (< 350 cells/μl) and four for clinical reasons. Among these 79, 41 (52%) started ART during study follow-up. Based on a CD4 cell count of 500 cells/μl, 129 (63%) would have become eligible for ART.

**Table 1:** Selected characteristics of study participants at enrolment (*n* = 206)

*Demographic*	
Sex, female [*n* (%)]	178 (86)
Age (years) [median (IQR)]	34 (28, 43)
Enrolment CD4 count [*n* (%)]	
≤ 559 cells/μL	53 (26)
560–632 cells/μL	51 (24)
633–768 cells/μL	53 (26)
> 768 cells/μL	49 (24)
Current marital status [*n* (%)]	
Never married	163 (79)
Monogamous marriage	27 (13)
Polygamous marriage	3 (1)
Separated/divorced/widowed	13 (6)
Religion [*n* (%)]	
None	15 (7)
Shembe	39 (19)
Zionist	62 (30)
Western Christian	78 (38)
Other	12 (6)
Education, achieved matriculation or higher [*n* (%)]^a^	54 (27)
Parity [median (IQR)]^b^	2 (2, 4)
Number of lifetime partners [*n* (%)]	
1	28 (14)
2–4	128 (62)
≥5	50 (24)
Number of children [median (IQR)]^c^	3 (1, 4)
Employment [*n* (%)]	
Currently employed	42 (20)
Ever employed	108 (52)
Receiving social welfare grant [*n* (%)]	150 (73)
Household unable to afford food in past 12 months [*n* (%)]	44 (21)
Always resident in Africa Centre DSA [*n* (%)]^d^	86 (42)
Stigma score [median (IQR)]^e^	42 (36,48)
Gender norms score [median (IQR)]^f^	39 (37, 43)
Taken nutritional supplements in first 6 months of study [*n* (%)]	23 (11)
*Social capital*	
Neighbour would contribute time for a project [*n* (%)]	149 (72)
Neighbour would contribute money (R10) for a project [*n* (%)]	138 (67)
Who would deal with a problem that affected the entire neighbourhood? [*n* (%)]	
Municipal/district leaders	46 (22)
Each person/household individually or neighbours	14 (7)
Traditional leaders	74 (36)
Traditional and municipal/district leaders would act together	72 (35)
Community groups in neighbourhood, yes [*n* (%)]	156 (76)
How much can you rely on family/friends if you have a serious problem? [*n* (%)]	
A little	22 (11)
A lot	179 (87)
Not at all	5 (2)
How often do you spend time with neighbours? [*n* (%)]	
Every day	65 (32)
Several days a week	53 (26)
At least once a fortnight	8 (4)
Once a month	24 (12)
Less than once a month/not at all	56 (27)

a Data missing for eight patients.

b Female patients only; data missing for five patients.

c Male patients only.

d Data available for *n* = 170. In the Africa Centre surveillance system, household membership is not conditional on residency; an individual can be recorded as a nonresident household member if they are residing in a household outside the demographic surveillance area (DSA) but remain socially connected to a household in the DSA. Changes in residence by individuals and households within the DSA (internal migration) and into or out of the DSA (external migration) have been documented since January 2000.

e HIV stigma was measured by a set of 24 questions adapted from Sayles *et al*.’s 28-item scale [11]. The questions assessed the individual’s perceived HIV stigma in the community and internalized HIV stigma. Higher stigma score represents greater HIV-related stigma. The maximum possible stigma score was 72 and the minimum was 24.

f Gender norms were measured by a set of 19 questions from 24 questions developed by Pulerwitz *et al*. [12]. Although the Pulerwitz *et al*. gender norms scale was originally administered to men only, on review it was considered an appropriate measure of gender norms for both sexes and administered to men and women. Scores could range between 19 and 57, with higher scores indicating more equitable gender norms and lower scores indicating male-dominant gender norms.

DSA, demographic surveillance area; IQR, interquartile range.

Univariably, the following were significantly associated with increased hazard of becoming ART-eligible: male sex [hazard ratio (HR) 3.13; 95% confidence interval (CI) 1.82–5.39; *P* < 0.001], older age [reference ≤ 30 years; 31–40 years, HR 2.16 (95% CI 1.24–3.75); ≥ 41 years, HR 1.55 (95% CI 0.88–2.76); *P* = 0.02], self-report of household going without food in the previous 12 months (HR 1.58; 95% CI 0.99–2.54; *P* = 0.057), higher number of lifetime partners [reference 1 partner; 2–4 partners, HR 1.33 (95% CI 0.63–2.83); ≥ 5 partners, HR 2.40 (95% CI 1.08–5.33); *P* = 0.032], lower enrolment CD4 cell count [reference ≤ 559 cells/μl; 560–632 cells/μl, HR 0.73 (95% CI 0.42–1.26); 633–768 cells/μl, HR 0.41 (95% CI 0.22–0.75); > 768 cells/μl, HR 0.20 (95% CI 0.09–0.45); *P* < 0.001], taking nutritional supplements between enrolment and 6 months (HR 2.06; 95% CI 1.11–3.83; *P* = 0.022) and ever use of recreational drugs (HR 2.35; 95% CI 1.08–5.15; *P* = 0.03) (Table 2). Once adjusted for sex, the number of lifetime partners and ever use of recreational drugs were no longer significant. Reported ever alcohol use was 35% among female patients and 89% among male patients (*P* < 0.001), and reported use in the first 6 months of follow-up was 4% and 43% among female and male patients, respectively. Neither ever or current alcohol use was associated with time to ART eligibility. The limited number of female current drinkers hindered exploration of associations between time to ART eligibility and alcohol use in a model including male sex.

**Table 2:** Multivariable Cox proportional hazards regression analysis of factors associated with time to antiretroviral therapy (ART) eligibility

Variable	HR	95% CI	aHR	95% CI	*P* value
Enrolment CD4 cell count					
≤559 cells/μL	1		1		
560–632 cells/μL	0.73	0.42–1.28	0.46	0.25–0.83	
633–768 cells/μL	0.41	0.22–0.75	0.30	0.16–0.57	
>768 cells/μL	0.20	0.09–0.45	0.17	0.08–0.38	<0.001
Sex					
Female	1		1		
Male	3.13	1.82–5.39	4.00	2.20–7.28	<0.001
Household unable to afford food in past 12 months					
No	1		1		
Yes	1.58	0.99–2.54	1.58	0.98–2.54	0.062
Taken nutritional supplements in first 6 months of study					
No	1		1		
Yes	2.06	1.11–3.83	2.38	1.26–4.48	0.007

HR, hazard ratio; aHR, adjusted hazard ratio; CI, confidence interval.

Multivariably, lower CD4 cell count at enrolment, being male, residing in a household with food shortage in the last year, and nutritional supplement use remained significantly associated with increased hazard of meeting ART eligibility criteria (Table 2). There was no evidence of an interaction between enrolment CD4 cell count and sex (*P* = 0.64). The proportional hazards assumption for the final model was met (global test *P* value = 0.81). Median time to ART eligibility at a CD4 cell count < 350 cells/μl was 12.0 months (95% CI 8.3–25.1 months) for men, and 33.9 months (95% CI 27.0–36.8 months) for women. The overall rate of progression to eligibility at a CD4 cell count < 350 cells/μl was 25.6 per 100 person-years (PY) (95% CI 20.5–31.9 per 100 PY); rates by year of follow-up were: 19.3 (95% CI 13.8–26.8) per 100 PY for the first year, 33.6 (95% CI 23.9–47.3) per 100 PY for the second year and 31.8 (95% CI 16.5–61.1) per 100 PY for the third year. Median time to CD4 cell count < 500 cells/μl for men was 8.3 months (95% CI 6.0–18.6 months) and for women it was 17.8 months (95% CI 15.9–19.7 months).

## Discussion

Just over one in three HIV-infected adults enrolled in HIV care at an early stage became eligible for ART at a CD4 cell count < 350 cells/μl. In these HIV-infected people with an initial CD4 count > 500 cells/μl, progression to ART eligibility was associated with lower baseline CD4 cell count, male sex, household food insecurity and the personal use of nutritional supplements.

The study population was predominantly female, consistent with previously reported data [9],[13],[14], and probably reflecting higher population-level CD4 cell counts among women [15] and the different entry points into HIV care. Previous work did not find any association between sex and disease progression pre-ART initiation [4]; however, mortality after ART initiation is consistently higher in men [16]. Men enrolled in our study may be incompletely representative of all men with CD4 cell counts > 500 cells/μl as a consequence of unmeasured clinical characteristics, but our finding that time to ART eligibility was significantly shorter for men highlights the need to develop male-oriented strategies throughout HIV care [17],[18]. Current guidelines recommend ART for all pregnant females and individuals with TB disease, regardless of CD4 cell count, but this was not the case throughout the study period. We did not estimate the effect this might have on overall progression to eligibility, although the estimates would be unlikely to change substantially as the majority of TB cases and pregnancies are associated with CD4 cell counts < 350 cells/μl in South Africa [19],[20].

Use of nutritional supplements in the first 6 months of follow-up may indicate poorer nutritional state and low body mass index (BMI); similarly, the association with self-reported household lack of food could also reflect a link between food insecurity, malnutrition and disease progression. Other household socioeconomic status indicators were not associated with time to ART eligibility, suggesting a specific role of nutrition [21]. However, there is no clear evidence that macronutrient or micronutrient supplementation delays HIV disease progression in nonpregnant adults 22–24. The complex interplay between HIV and nutrition means that determining cause and effect relationships is difficult; this was certainly the case in this analysis, given the study design and the lack of clinical indicators such as BMI [25]. There was no evidence of an association between disease progression and any behavioural factors (including ART knowledge, social capital, social support, relationship characteristics, and internalized stigma). Therefore, while interventions to address these issues may have merit, they should not be expected to delay disease progression.

Our results are similar to those from a smaller study, also from KwaZulu-Natal, of female patients monitored from HIV seroconversion, where 43% reached a CD4 cell count of < 350 cells/μl within 2 years [26]. Although the individual-level benefit/risk ratio of commencing ART at CD4 cell counts > 350 cells/μl has not yet been established [27],[28], international and national guidelines now recommend initiation of ART if the CD4 cell count is < 500 cells/μl [29],[30]. Whereas immediate ART may improve survival in adults with CD4 cell counts > 500 cells/μl, this benefit might be reversed by potentially high rates of treatment failure or disengagement from care [31]; the latter may be more likely as programmes expand 32–34. In this population, around one in four adults now present for HIV care with a CD4 cell count > 500 cells/μl [7]; our finding that most of such adults had not become eligible during follow-up is consistent with regional natural history data [35] and suggests that risks of treatment failure and costs of treatment could be deferred for a considerable time. However, whether or not this changes the overall individual benefit/risk ratio and whether there are additional population-level risks or benefits remain to be seen [36],[37]. Our data would suggest that many individuals will become eligible within one or two visits in pre-ART care, especially if visits are less frequent than the recommended 6 months [13]. Education and counselling should emphasize the importance of retention in care to all patients not yet requiring ART. Focused messages and systems may be required to enable retention of men, who may have faster disease progression and poorer retention in pre-ART care [13],[38].
